# Nonusage Attrition of Adolescents in an mHealth Promotion Intervention and the Role of Socioeconomic Status: Secondary Analysis of a 2-Arm Cluster-Controlled Trial

**DOI:** 10.2196/36404

**Published:** 2022-05-10

**Authors:** Laura Maenhout, Carmen Peuters, Greet Cardon, Geert Crombez, Ann DeSmet, Sofie Compernolle

**Affiliations:** 1 Department of Movement and Sports Sciences Ghent University Ghent Belgium; 2 Research Foundation Flanders (FWO) Brussels Belgium; 3 Department of Experimental-Clinical and Health Psychology Ghent University Ghent Belgium; 4 Faculty of Psychology and Educational Sciences Université Libre de Bruxelles Brussels Belgium; 5 Department of Communication Studies University of Antwerp Antwerp Belgium

**Keywords:** mHealth, nonusage attrition, adolescents, socioeconomic status, mobile phone

## Abstract

**Background:**

Mobile health (mHealth) interventions may help adolescents adopt healthy lifestyles. However, attrition in these interventions is high. Overall, there is a lack of research on nonusage attrition in adolescents, particularly regarding the role of socioeconomic status (SES).

**Objective:**

The aim of this study was to focus on the role of SES in the following three research questions (RQs): When do adolescents stop using an mHealth intervention (RQ1)? Why do they report nonusage attrition (RQ2)? Which intervention components (ie, self-regulation component, narrative, and chatbot) prevent nonusage attrition among adolescents (RQ3)?

**Methods:**

A total of 186 Flemish adolescents (aged 12-15 years) participated in a 12-week mHealth program. Log data were monitored to measure nonusage attrition and usage duration for the 3 intervention components. A web-based questionnaire was administered to assess reasons for attrition. A survival analysis was conducted to estimate the time to attrition and determine whether this differed according to SES (RQ1). Descriptive statistics were performed to map the attrition reasons, and Fisher exact tests were used to determine if these reasons differed depending on the educational track (RQ2). Mixed effects Cox proportional hazard regression models were used to estimate the associations between the use duration of the 3 components during the first week and attrition. An interaction term was added to the regression models to determine whether associations differed by the educational track (RQ3).

**Results:**

After 12 weeks, 95.7% (178/186) of the participants stopped using the app. 30.1% (56/186) of the adolescents only opened the app on the installation day, and 44.1% (82/186) stopped using the app in the first week. Attrition at any given time during the intervention period was higher for adolescents from the nonacademic educational track compared with those from the academic track. The other SES indicators (family affluence and perceived financial situation) did not explain attrition. The most common reasons for nonusage attrition among participants were perceiving that the app did not lead to behavior change, not liking the app, thinking that they already had a sufficiently healthy lifestyle, using other apps, and not being motivated by the environment. Attrition reasons did not differ depending on the educational track. More time spent in the self-regulation and narrative components during the first week was associated with lower attrition, whereas chatbot use duration was not associated with attrition rates. No moderating effects of SES were observed in the latter association.

**Conclusions:**

Nonusage attrition was high, especially among adolescents in the nonacademic educational track. The reported reasons for attrition were diverse, with no statistical differences according to the educational level. The duration of the use of the self-regulation and narrative components during the first week may prevent attrition for both educational tracks.

**Trial Registration:**

ClinicalTrials.gov NCT04719858; http://clinicaltrials.gov/ct2/show/NCT04719858

## Introduction

Mobile health (mHealth) interventions seem promising for behavior change [1-6]. mHealth is a part of the broad category of digital health interventions and is defined as the support of health practices through mobile devices, such as mobile phones, patient monitoring devices, PDAs, and other wireless devices [7]. mHealth offers the opportunity to reach a large part of the population in a tailored, cost-effective manner [2,8-11]. Despite its potential, many mHealth interventions report trivial-to-small effects or effects that are not sustained in the long term [1,2,9,12-14]. Evidence suggests that this is partly because of low levels of adherence and high nonusage attrition rates, which are common in digital health interventions [15-19]. Nonusage attrition refers to participants who stop using the digital intervention, although they could still be participating in the research protocol (eg, filling out questionnaires) [18]. Nonusage attrition to commercial apps used in real-world settings reaches an average rate of 62%, with 21% of users abandoning an app after the first use [20]. Nonusage attrition to research-based mHealth interventions ranges from 32% to 75%, often depending on how long an intervention lasts and whether a study occurs in a real-world rather than controlled context [21-24].

Unfortunately, most research on this topic has focused on adults. There is a dearth of research on attrition rates of adolescents, although there has been a sharp increase in the use of digital interventions for behavior change within this age group [14,25]. A notable exception is the study by Egilsson et al [26], who developed the social health game *SidekickHealth*. This app focuses on three health categories: food and drink intake, physical activity, and mental health. Young people can set goals and complete missions (ie, gamification) both individually and in small groups. Attrition rates were reviewed weekly to check whether adolescents completed at least three health exercises within the app. During their pilot study among Icelandic adolescents aged between 15 and 16 years, the authors reported a nonusage attrition rate of 35% from initiation to the 6-week follow-up. The average frequency of completing in-app health exercises decreased significantly in the first week (from an average of 55.25 to 13.63 exercises), notwithstanding the large effort to keep the app entertaining and fun (eg, by adding a reward system and storyline highlighting progress) [26].

Various behavior change techniques are typically used in mHealth interventions [1,27-29], among which are goal setting and self-monitoring (ie, self-regulation techniques) [1,27,29,30]. Similarly, other techniques are required, not necessary to foster behavior change, but rather engagement (eg, a reward system). Research indicates that when adolescents are more engaged, there is a reduced risk of attrition, leading to a higher probability of intervention effectiveness [22,31]. In this regard, it has been suggested that narratives (ie, stories that portray human thought and action with a beginning, middle, and end) [32-34] and chatbots [35] might increase user engagement with digital health interventions. These intervention components can be of particular interest to adolescents from lower socioeconomic status (SES), as this group tends to have lower digital health literacy [36]. Narratives are less language demanding, and chatbots provide the opportunity to replace researchers offering direct communication during a study, which could mitigate the problems of health literacy because participants can ask questions based on their own use of language [37,38].

Special efforts to engage adolescents from lower SES backgrounds are needed, as these adolescents tend to have lower health outcomes than those from higher SES groups [39-45]. It further appears that digital health tools are currently only used to a small extent by people with low SES [37,46], although many of them do own a smartphone (eg, smartphone ownership of Flemish adolescents of all different socioeconomic backgrounds amounts to 93% [47]). Furthermore, digital interventions do not show equivalent efficacy for people of low and high SES, meaning that there is no evidence that digital interventions are effective for people with low SES, whereas this appears to be the case for their higher SES counterparts [36]. The fact that high-SES groups engage more with digital tools and that they prove to be effective only for them may further widen the health gap between higher and lower SES groups [46]. Past studies have consequently recommended adapting interventions to adolescents with lower SES [36,39-41,45,48]. However, no studies have investigated the SES differences in nonusage attrition among adolescents in mHealth interventions and whether intervention components aimed at increasing engagement also effectively lead to longer use of the intervention in this target group.

To counteract small intervention effects (Cohen *d*=0.22 in mHealth interventions for youth [2]) or prevent only the short-term use of mHealth interventions for adolescents, it is necessary to further identify when and why adolescents stop using an intervention (RQ1 and RQ2). Within this context, it is also important to investigate the intervention components that positively impact attrition (RQ3). All RQs also examine whether the results differ according to SES, as engaging vulnerable groups is key to tackling socioeconomic health inequalities [46].

## Methods

### Study Design

This study concerns secondary analyses of a larger 2-arm cluster-controlled trial that evaluated the effectiveness of the #LIFEGOALS intervention. A total of 6 schools with 223 participants were assigned to the intervention group and 5 schools with 118 participants were assigned to the control group. The intervention group received the #LIFEGOALS intervention to promote a healthy lifestyle for 12 weeks (ie, 85 days). The control group received no intervention. A more detailed description of the study is provided in the flowchart in Multimedia Appendix 1. In this paper, only data from the intervention group will be described, as the focus is on nonusage attrition with the app. Written informed consent was obtained from the participants and their parents before participation in the study.

### Ethics Approval

This study was approved by the Ethical Committee of the Faculty of Psychology and Educational Sciences of Ghent University (2020/2070 Laura Maenhout).

### Participants and Recruitment

Participants were recruited through schools via convenience sampling in August and September 2020. The inclusion criteria were adolescents of the seventh, eighth, or ninth grade of general education. The exclusion criteria were schools of special needs education and education for nonnative speakers (in preparation for regular education). A total of 27 Flemish schools were contacted via email to participate in this study. When the schools did not respond within 2 weeks, they were contacted by phone. Of the 27 schools, 12 (ie, response rate 44%) agreed to participate in the study. To allow for clustering in the analysis of the effect study, a target number of 30 adolescents per school was set. The school selected the classes, but the researchers actively monitored that there was an equal mix of grades and educational tracks (academic vs nonacademic) across the intervention and control groups. Because of the COVID-19 measures, of the 6 schools, 1 (17%) withdrew from the intervention group, resulting in 1 school from the control group being assigned to the intervention group. This resulted in an overrepresentation of adolescents in the academic track (114/186, 61.3%) than in the nonacademic track (72/186, 38.7%) in the intervention group. The researchers sent information letters and informed consent for both adolescents and parents to the school contact person by mail in advance. The contact person distributed informed consent to the participating classes, with the intention that both signed consent forms could be collected at the first class visit. Adolescents who provided both signed consent forms during the first class visit were rewarded with a power bank. Adolescents who lacked one of the consent forms were encouraged to have the forms signed by the second class visit. If adolescents submitted their consent forms during subsequent class visits, they could still participate and receive their power bank. In addition, cinema tickets (ie, incentive at the end of the intervention period) were never distributed if consent forms had not yet been submitted. Finally, adolescents for whom consent forms were still missing at the end of the intervention period were removed from the data (21/186, 11.3%).

### Intervention

#LIFEGOALS is an mHealth intervention developed for Flemish adolescents aged between 12 and 15 years to improve their mental health by promoting healthy lifestyle behaviors (ie, sufficient sleep and physical activity, daily breakfast intake, and sedentary behavior reduction) [45,49-52]. #LIFEGOALS is theory-based (ie, based on the Health Action Process Approach [53], Elaboration Likelihood Model [54], and Persuasive Systems Design [55]) and developed in close collaboration with target users and stakeholders. In total, 249 adolescents were involved during intervention development [56]: adolescents’ views on a health app and chatbot were identified through focus group discussions (112/249, 44.9%); a class of adolescents was involved in developing and filming of the narrative (47/249, 18.9%); prototypes of the app and chatbot were tested regularly to detect bugs (11/249, 4.4%); a steering committee was set up and consulted at various times throughout the process (14/249, 5.6%); and finally, a pilot study with process evaluation interviews was conducted in January 2020, after which final adjustments were made toward the effect study (65/249, 26.1%). #LIFEGOALS includes (1) a self-regulation component associated with Fitbit for goal settings, action planning, coping planning, monitoring, and providing feedback; (2) a narrative component (ie, every week participants receive a new episode [2-5 minutes] of a dedicated youth series made for this intervention) for modeling, attitude change, and increased engagement; and (3) a chatbot component (ie, a web-based coach that provides an automated answer to user questions and sends 2 encouraging messages per week) for social support and sustained engagement with the intervention [56]. In addition, information (eg, on the benefits of health behaviors and relevant [youth] health organizations for further information or support) and a reward system (in which coins can be earned to achieve goals, which the participants can then use to personalize their personal avatar) are included in the intervention. The participants were free to choose which lifestyle behaviors they wanted to target, and to what extent they wanted to use the app. A screenshot of the app can be found in Multimedia Appendix 2.

### Procedure

Three waves of data collection were conducted from October 2020 to May 2021. The first wave (intervention group, 67/186, 36%) began in October 2020, the second wave (55/186, 29.6%) in November 2020, and the third wave (64/186, 34.4%) in January 2021. The researchers visited the intervention schools 4 times. During the first school visit, adolescents received information about the project and were provided with an accelerometer (ie, Axivity [AX3; Axivity Ltd]), which they were instructed to wear for 1 week (beyond the scope of this study). They were also asked to complete a prequestionnaire including sociodemographic factors. During the next visit (1 week later), the accelerometers were retrieved, and temporary devices were provided to those without a smartphone or Fitbit (charge 2 or 3) for the duration of the study. Subsequently, the #LIFEGOALS app was installed on participants’ smartphones and connected with the associated Fitbit. The participants were asked to use the app for 12 consecutive weeks. Roll-up banners were set as cues in visible places (ie, in the classroom where adolescents were primarily taught) to encourage the app use (Multimedia Appendix 3). After 12 weeks, the participants completed the postquestionnaire and wore the accelerometers for another week. During the last visit (1 week later), accelerometers were retrieved and incentives (ie, cinema tickets) were provided to those who completed all the questionnaires and wore the accelerometer. To gain insights into when adolescents stopped using the app, their log data were monitored during the entire intervention period. To explore the reasons why participants stopped using the app, a web-based questionnaire was sent by text message (or by email for those not providing their phone number; 12/186, 6.5%) after a participant had not used the app (including narrative and chatbot) for 3 weeks. Participants who indicated in the web-based questionnaire that they still had the intention to use the app were not asked further questions but were contacted again when they had not used the app for another 3 weeks. Participants who did not complete the questionnaire were considered nonusers without any information about their attrition reasons. Participation in the web-based questionnaire was encouraged by reminding adolescents of cinema tickets via SMS text messaging.

### Measures

#### General Sociodemographic Information

Sociodemographic information was reported by the adolescents themselves, including gender (boy, girl, or other), age (date of birth), language spoken at home (Dutch, French, Turkish, Arabic, English, or other), grade (seventh, eighth, or ninth), and SES. All items were answered by the adolescents themselves. Various indicators were used to measure SES, as currently no consensus is reached in the literature on the most appropriate indicator [57]. First, adolescent’s educational track was asked. For the seventh and eighth grades, response options were A track (ie, mainstream education) or B track (ie, for academically less-performing students that prepares them for vocational education), and for the ninth grade, response options were general academic, technical, vocational, or arts education. The 3 grades were subsequently recoded into academic and nonacademic tracks. It is hypothesized that adolescents from the nonacademic track would have lower SES compared with adolescents from the academic track, analogous to the Flemish Health Behavior in School-Aged Children (HBSC) questionnaire [58]. Second, the educational level of both the father and mother (not applicable, I do not know, no diploma, primary school [until aged 12 years], secondary school [until aged 18 years], high school or nonuniversity, or university) was assessed. Third, family affluence was estimated using the Family Affluence Scale (FAS) III. This scale is an international indicator of adolescents’ SES used in the HBSC questionnaire [59] and is defined as a socioeconomic proxy for family wealth [60]. This scale has been widely used [61] and validated alongside other SES measures (eg, parental occupation) and objective measures of country wealth (eg, per-capita income and gross domestic product) [60,62,63]. The FAS III consists of the following six items [60]: *Does your family own a car or another motorized vehicle?* (No=0; Yes, one=1; Yes, 2 or more=2), *Do you have your own bedroom?* (No=0; Yes=1), *How many computers (including laptops and tablets, not including game consoles and smartphones) does your family own?* (None=0; One=1; Two=2; More than two=3), *How many bathrooms (room with a bath/shower or both) are there in your home?* (None=0; One=1; Two=2; More than two=3), *Does your family have a dishwasher?* (No=0; Yes=1), and *How many times did you and your family travel out of Belgium for holiday/vacation last year?* (Never=0; Once=1; Twice=2; More than twice=3). A composite FAS score (ranging from 0 to 13) was calculated for each adolescent based on their responses to these 6 items [59]. Consequently, three groups were created according to the cutoff points of the Flemish HBSC questionnaire (0-7=low FAS score, 8-11=medium FAS score, and 12-13=high FAS score) [64]. Finally, the perceived financial situation was measured using the following question: *How easily can your family pay for everything you need in a month (eg, food, rent, things for school, and so on)?* of the Flemish Youth Research Platform (Jongeren Overleg Platform School Monitor 2018 [65]). Answer options were rated on a 6-point Likert scale ranging from very difficult to very easy. A total of two categories were created based on the median (1=very difficult to quite easy and 2=easy to very easy). These 4 SES measures referred both to the level of education, which can be seen as an indication of certain knowledge and skills and to material prosperity (ie, the FAS and perceived financial situation). Moreover, several dimensions can be distinguished in different SES measures: the adolescent, the parents, and the whole family.

#### Log Data Variables

Log data of the #LIFEGOALS app to measure (1) nonusage attrition and (2) the use of the three intervention components (ie, self-regulation, narrative, and chatbot) were stored on the cloud server of Ghent University, Department of Information and Communication Technology. Nonusage attrition was operationalized as the number of days from the start of the intervention (ie, the day adolescents installed the app on their smartphone) to the last day that the app activity was recorded. For the use of the 3 intervention components, the duration (in minutes) participants spent using the self-regulation component, watching the narrative, and interacting with the chatbot during the first week was extracted. The duration started when one of the app components (ie, self-regulation component, narrative, and chatbot) was clicked and ended when the adolescent called up another app component, left the app, or if the smartphone went into sleeping or inactive mode. As the self-regulation component consisted of several elements (eg, goal setting [Set Mission], coping planning [Tools], self-monitoring [Graphs], and agenda [My Agenda]), the sum score of the time spent on all these elements was calculated.

#### User-Reported Reasons for Attrition

A total of 14 items were formulated based on the literature [18,66-68] and discussions with the research team. Participants indicated whether they agreed with the reason for attrition on a 5-point Likert scale, ranging from strongly disagree to strongly agree. Furthermore, participants were free to give another reason for not using the #LIFEGOALS app anymore via an open answer option. Finally, they were asked whether they would recommend the app to their friends (yes, no, or not applicable).

### Analysis

Descriptive statistics were provided for participants’ characteristics and reasons for nonusage attrition. Survival analysis (ie, Kaplan-Meier plots and logrank test statistics) [69,70] was used to estimate the time to attrition and assess statistically significant differences among the SES groups (RQ1). The number of days between the start of the intervention (ie, the day adolescents installed the app on their smartphone) and the last day of app use was the time variable, and the event variable was specified as attrition before the end of the 12-week (ie, 85 days) intervention. Cases were classified as censored when the app was still being used by the end of the 12-week intervention period. To test for significant differences in participant characteristics between responders and nonresponders in the attrition questionnaire, an independent samples 2-tailed *t* test was used for the continuous variable (ie, age) and chi-square tests were conducted for categorical variables (ie, gender, grade, and type of education). Fisher exact tests were used to determine if there were significant differences between attrition reasons and educational tracks (RQ2). Therefore, the 14 variables with attrition reasons were recoded into variables with two categories: strongly disagree-neutral and agree-strongly agree. Mixed effects Cox proportional hazard regression models with clusters (ie, classes) as a random factor [71] were used to identify whether the duration of the self-regulation, narrative, or chatbot component during the first week could predict attrition (RQ3). It was chosen to run the models with clusters to control for the random effects of the class in which each adolescent was nested (eg, the attention given to the project by the teacher). However, given the correlation between *class* and *educational track* of the adolescent, the standard Cox proportional hazard regression model was also run as sensitivity analysis (Multimedia Appendix 4). First, single-predictor models were fitted for each potential confounding variable (ie, age, grade, gender, home language, educational track, family affluence, and perceived financial situation) and for the duration in the different intervention components (ie, self-regulation component, narrative, and chatbot) during the first week. Second, a multiple-predictor model was fitted with the significant confounding variables from the previous step and the duration of each intervention component during the first week. Finally, an interaction term was added to the fully adjusted multiple-predictor model to test whether the associations between the duration of each intervention component in the first week and nonusage attrition differed among SES groups. Statistical analyses were performed using the coxme package in R (version 4.1.0; R Foundation for Statistical Computing). An *α* level of .05 was used to assess the statistical significance.

## Results

### Participant Characteristics

In total, the intervention group consisted of 186 adolescents (ie, participation rate, 83%). The characteristics of the sample are presented in Table 1. The log data related to duration in the different components during the first week showed that there were large differences among the participants in terms of use duration, but most of the adolescents hardly spent any time in the app, with a median of 1.41 minutes per week for the self-regulation component, 0.03 minutes per week for the narrative, and 0.39 minutes per week for the chatbot.

**Table 1:** Participant characteristics of the #LIFEGOALS intervention group (n=186).

Sociodemographic characteristic		Value
Age (years), mean (SD; range)		13.51 (0.96; 11.83-15.66)
**Gender, n (%)**		
	Adolescent male	90 (48.4)
	Adolescent female	94 (50.5)
	Other	2 (1.1)
**Home language, n (%)**		
	Dutch	148 (79.6)
	French	6 (3.2)
	Turkish	10 (5.4)
	Arabic	8 (4.3)
	English	1 (0.5)
	Other	13 (7)
**Grade, n (%)**		
	Seventh	67 (36)
	Eighth	60 (32.3)
	Ninth	59 (31.7)
**Type of education, n (%)**		
	Academic track	114 (61.3)
	Nonacademic track	72 (38.7)
**Educational degree of the father, n (%)**		
	Not applicable	4 (2.2)
	I do not know	110 (59.1)
	No diploma	1 (0.5)
	Primary school (until 12 years of age)	3 (1.6)
	Secondary school (until 18 years of age)	24 (12.9)
	High school, nonuniversity	21 (11.3)
	University	23 (12.4)
**Educational degree of the mother, n (%)**		
	Not applicable	2 (1.1)
	I do not know	101 (54.3)
	No diploma	4 (2.2)
	Primary school (until 12 years of age)	4 (2.2)
	Secondary school (until 18 years of age)	17 (9.1)
	High school, nonuniversity	35 (18.8)
	University	23 (12.4)
**Family affluence, mean (SD; range)**		9.09 (2.03; 2-13)
	Low FAS^a^ score, n (%)	38 (20.4)
	Medium FAS score, n (%)	128 (68.8)
	High FAS score, n (%)	20 (10.8)
**Perceived financial situation, n (%)**		
	Very difficult	0 (0)
	Difficult	5 (2.7)
	Quite difficult	3 (1.6)
	Quite easy	30 (16.1)
	Easy	92 (49.5)
	Very easy	56 (30.1)
**Log data–derived variables (in minutes), median (IQR; range)**		
	Duration of self-regulation during the first week	1.41 (5.36; 0-34.21)
	Duration of narrative during the first week	0.03 (0.77; 0-16.35)
	Duration of engaging with the chatbot during the first week	0.39 (2.52; 0-43.33)

^a^FAS: Family Affluence Scale.

### Attrition Patterns

**Figure 1:**
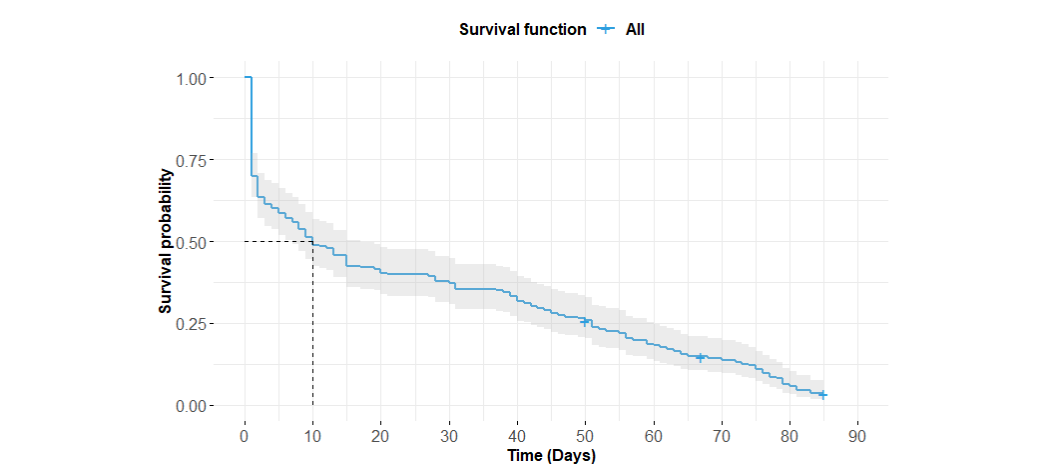
Attrition pattern of the #LIFEGOALS intervention.

The attrition pattern of the entire 12-week study period is presented by the Kaplan-Meier plot in Figure 1. Across the study period, there was a 4.3% (8/186) completion rate, with the remaining 95.7% (178/186) of the participants stopping the use of the app before the end of the study. The median survival time was 10 (95% CI 7-17) days. Of the 186 adolescents, 56 (30.1%) only opened the app on the installation day (ie, day 1) and 82 (44.1%) stopped using the app in the first week.

**Figure 2:**
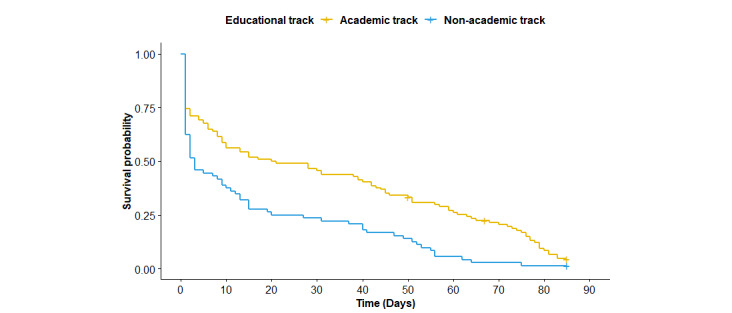
Kaplan-Meier plots according to socioeconomic status indicator (educational track).

**Figure 3:**
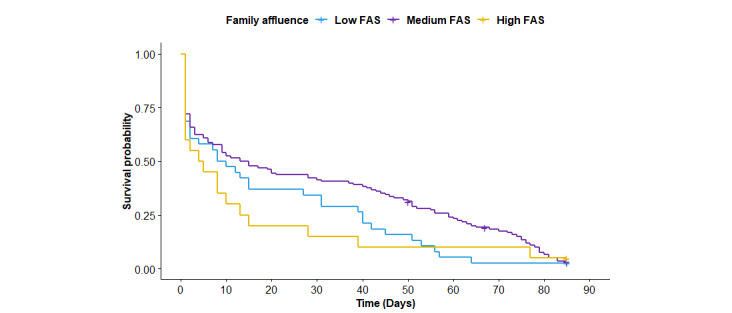
Kaplan-Meier plots according to socioeconomic status indicator. FAS: Family Affluence Scale.

**Figure 4:**
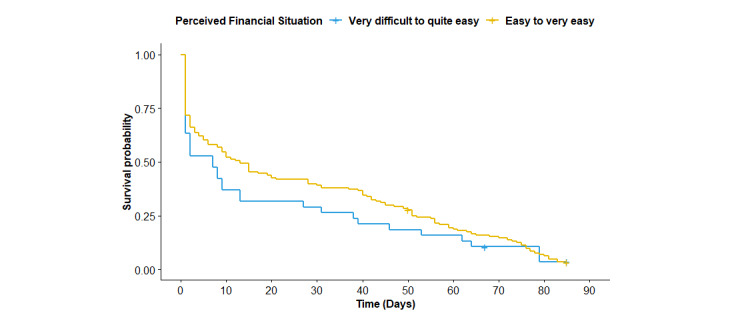
Kaplan-Meier plots according to socioeconomic status indicator (perceived financial situation).

Next, we examined whether the attrition rate differed according to SES indicators included in the study. Because more than half of the adolescents reported not knowing the degree of education of their fathers (110/186, 59.1%) and/or mothers (101/186, 54.3%), the difference in the attrition rate based on this indicator was not examined. Figures 2-4 show the Kaplan-Meier plots according to (1) educational track, (2) family affluence, and (3) perceived financial situation. According to the logrank tests (Table 2), only the educational track showed a significant difference (*P*<.001), meaning that attrition at any given time during the intervention period was significantly higher for adolescents from the nonacademic track compared with the academic track.

**Table 2:** Logrank tests according to socioeconomic status (SES) indicators.

SES indicator	Logrank value (χ^2^) (*df*)	*P* value
Educational track	16.7 (1)	*<.001* ^a^
Family affluence	5.2 (2)	.07
Perceived financial situation	1.3 (1)	.3

^a^Italicization indicates *P*<.05.

### Reasons for Nonusage Attrition

Of the 186 adolescents, 175 (94.1%) received the attrition questionnaire at least once during the intervention period (meaning they had not used the app for 3 weeks). Finally, 25.1% (44/175) of adolescents completed an attrition questionnaire. Table 3 shows the participant characteristics of receivers, responders, and nonresponders. There was a significant difference between responders and nonresponders according to the educational track, with more adolescents from the academic track answering the attrition questionnaire than adolescents from the nonacademic track (*P*=.046).

**Table 3:** Participants’ characteristics with regard to the attrition questionnaire.

Sociodemographic characteristic		Values				Significance of difference				
		Receivers (n=175)	Responders (n=44)	Nonresponders (n=131)	*t* test (*df*)^a^		Chi-square (*df*)		*P* value	
Age (years), mean (SD; range)		13.42 (0.97; 11.83-15.66)	13.55 (0.94; 11.96-15.47)	13.45 (0.98; 11.83-15.66)	−0.55 (173)		N/A^b^		.59	
**Gender, n (%)**						N/A		2.60 (1)		.11
	Adolescent male	86 (49.1)	17 (38.6)	69 (52.7)						
	Adolescent female	87 (49.7)	27 (61.4)	60 (45.8)						
	Other	2 (0.2)	0 (0)	2 (1.5)						
**Grade, n (%)**						N/A		1.68 (2)		.43
	Seventh	66 (37.7)	13 (29.5)	53 (40.5)						
	Eighth	57 (32.6)	16 (36.4)	41 (31.3)						
	Ninth	52 (29.7)	15 (34.1)	37 (28.2)						
**Type of education, n (%)**						N/A		3.97 (1)		.*046*^c^
	Academic track	105 (60)	32 (72.7)	73 (55.7)						
	Nonacademic track	70 (40)	12 (27.3)	58 (44.3)						

^a^Independent samples 2-tailed *t* test.

^b^N/A: not applicable.

^c^Italicization indicates *P*<.05.

The most common reasons for the nonusage attrition of the #LIFEGOALS app were (percentages from agree to strongly agree; Table 4) (1) My behavior did not change by using the app (24/44, 55%), (2) I did not like the app (17/44, 39%), (3) I already use other apps to track and/or improve my lifestyle (17/44, 39%), (4) I already live a sufficiently healthy life (16/44, 36%), and (5) I was not motivated by my environment to keep using the app (eg, at home and by friends; 15/44, 34%). There were no statistically significant differences in the attrition reasons depending on the educational track; only a borderline significant difference for the reason that there are other things in the adolescent’s life that they consider more important than their health (*P*=.08), where more adolescents from the nonacademic track had indicated this reason compared with those from the academic track. A comprehensive descriptive table of what the adolescents indicated per answer category for each attrition reason, including the division according to educational track, can be found in Multimedia Appendix 5.

In addition to the items included in the questionnaire, adolescents could also fill in their own reasons for no longer using the app. Other reasons given by adolescents were forgetting to use the app because of tight schedules with other things (5/44, 11%); not having enough storage on the smartphone (n=1); being more engaged with the Fitbit itself than with the app (n=1); feeling difficult to be motivated (n=1); feeling no intrinsic trigger to use the app compared with other apps (n=1); and using an app feels rather obligatory (eg, filling in a goal); therefore, preferring to work on their health *on their own* rather than using an app (n=2).

Of the 44 adolescents, 25 (57%) would not recommend the app to their friends and 19 (43%) would.

**Table 4:** Reasons why adolescents stopped using the #LIFEGOALS app and a test of significance according to the educational track (n=44).

I do not use the #LIFEGOALS app anymore because...	Strongly disagree to neutral, n (%)	Agree to strongly agree, n (%)	Significance of difference (*P* value, 2-tailed)^a^
The app takes too much time.	31 (70)	13 (30)	.30
I am not allowed to use my mobile phone much at home.	39 (89)	5 (11)	.99
I already live a sufficiently healthy life.	28 (64)	16 (36)	.99
There were technical problems with the app.	35 (80)	9 (20)	.41
The app was too complicated.	33 (75)	11 (25)	.14
I did not like the app.	27 (61)	17 (39)	.74
My behavior did not change by using the app.	20 (45)	24 (55)	.50
The app did not meet my expectations.	37 (84)	7 (16)	.65
My friends did not use the app either.	37 (84)	7 (16)	.37
I did not get enough reminders to use the app.	34 (77)	10 (23)	.24
I was not motivated by my environment to keep using the app (eg, at home and by friends).	29 (66)	15 (34)	.17
I already use other apps to track or improve my lifestyle (eg, Fitbit app).	27 (61)	17 (39)	.74
There are other things in my life I consider more important than my health.	37 (84)	7 (16)	.08
The chatbot often answered my questions incorrectly.	38 (86)	6 (14)	.53

^a^Fisher exact tests.

### Cox Proportional Hazard Regression Models

The results of both the single- and multiple-predictor mixed effects Cox proportional hazard regression models are presented in Table 5. As shown in the single-predictor models, no sociodemographic variables were significantly related to attrition, except educational track (*P*=.02). Conversely, the use duration in all 3 components during the first week was found to be significantly related to survival time. Subsequently, a multiple-predictor model was constructed in which the educational track was included as a confounding variable and the duration of all 3 components as independent variables. Significant predictors of attrition were duration in the self-regulation component during the first week (*P*<.001) and duration in the narrative component during the first week (*P*=.03). When adolescents used the self-regulation (hazard ratio 0.902, 95% CI 0.867-0.939) and narrative component (hazard ratio 0.924, 95% CI 0.858-0.994) more often during the first week, they were less likely to drop out 12 weeks later. The duration of the chatbot component during the first week did not contribute significantly to the overall model. Furthermore, the effect of duration in the 3 components during the first week on attrition was not significantly different according to SES (ie, educational track).

**Table 5:** Results of the clustered Cox proportional hazard regression models.

						Single-predictor models												Multiple-predictor models																					
						Coefficient (SE)				HR^a^ (95% CI)			*P* value			Without an interaction term															With an interaction term								
																Coefficient (SE)					HR (95% CI)				*P* value				Coefficient (SE)					HR (95% CI)				*P* value	
**Sociodemographic variables**																																							
	Age (in years)				0.057 (0.125)				1.059 (0.828-1.354)			.65			N/A^b^					N/A				N/A				N/A					N/A				N/A		
	**Gender (reference: adolescent male)**																N/A					N/A				N/A				N/A					N/A				N/A
			Adolescent female				−0.045 (0.192)		0.956 (0.656-1.393)			.81																											
			Other				0.754 (0.754)		2.126 (0.485-9.328)			.32																											
	**Grade (reference: seventh grade)**																N/A					N/A				N/A				N/A					N/A				N/A
			Eighth grade		−0.161 (0.362)				0.851 (0.419-1.730)			.66																											
			Ninth grade		−0.325 (0.333)				0.722 (0.376-1.388)			.33																											
	Home language (reference: Dutch)				0.361 (0.199)				1.435 (0.971-2.120)			.07			N/A					N/A				N/A				N/A					N/A				N/A		
	Educational track (reference: academic track)				0.555 (0.228)				1.742 (1.115-2.722)			*.02* ^c^			0.750 (0.211)					2.117 (1.399-3.202)				*<.001*				0.794 (0.262)					2.211 (1.324-3.695)				*.002*		
	**Family affluence (reference: low FAS^d^ score)**																N/A					N/A				N/A				N/A					N/A				N/A
		Medium FAS score		−0.244 (0.201)				0.784 (0.529-1.161)			.22																												
		High FAS score		0.241 (0.291)				1.272 (0.720-2.249)			.41																												
	Perceived financial situation				−0.225 (0.194)				0.798 (0.546-1.167)			.24			N/A					N/A				N/A				N/A					N/A				N/A		
**Log data–derived variables**																																							
	Duration of self-regulation during the first week				−0.109 (0.019)				0.897 (0.864-0.931)			*<.001*			−0.103 (0.021)					0.902 (0.867-0.939)				*<.001*				−0.097 (0.024)					0.907 (0.866-0.951)				*<.001*		
	Duration of narrative during the first week				−0.111 (0.039)				0.895 (0.828-0.966)			*.01*			−0.079 (0.037)					0.924 (0.858-0.994)				*.03*				−0.033 (0.046)					0.968 (0.885-1.058)				.47		
	Duration of engaging with the chatbot during the first week				−0.065 (0.029)				0.937 (0.885-0.993)			*.03*			0.006 (0.029)					1.007 (0.951-1.065)				.82				−0.022 (0.043)					0.979 (0.900-1.064)				.61		
**Interaction with socioeconomic status**						N/A				N/A			N/A			N/A					N/A				N/A														
	Duration of self-regulation during the first week—educational track (reference: academic track)																											−0.011 (0.046)					0.989 (0.904-1.082)				.81		
	Duration narrative during first week—educational track (reference: academic track)																											−0.102 (0.081)					0.903 (0.770-1.060)				.21		
	Duration chatbot during first week—educational track (reference academic track)																											0.048 (0.059)					1.049 (0.935-1.177)				.41		

^a^HR: hazard ratio.

^b^N/A: not applicable.

^c^Italicization indicates *P*<.05.

^d^FAS: Family Affluence Scale.

## Discussion

### Principal Findings

This study investigated when and why adolescents stop using an mHealth intervention (RQ1 and RQ2) and explored whether the use duration of specific intervention components during the first week can predict attrition (RQ3). All RQs examined whether this differed according to SES.

Although mHealth interventions can be seen as potentially revolutionary, we are still in the age of promise rather than delivery [72]. One of the main challenges that still lies ahead is low adherence to and engagement with mHealth interventions [15-19,72]. Despite attempts to increase adherence and engagement in the current intervention (ie, participatory development, adding a narrative and chatbot, and reward system), the results of the #LIFEGOALS intervention showed that 95.7% (178/186) of the participants stopped using the app before the end of the study period. These numbers are high compared with the attrition rates obtained by other research-based mHealth interventions (ie, 32%-75%) [21-24]. Although most of these studies focused on adults, the study by Egilsson et al [26], focusing on adolescents, also reported a much lower attrition rate (ie, an attrition rate of 35% after 6 weeks). A possible explanation for our higher rates than those reported by Egilsson et al [26] might be the difference in recruitment strategy; in this study, whole classes were recruited in which all pupils were asked to participate during a class visit, whereas in the study by Egilsson et al [26], an email was sent via school officials to parents and legal guardians asking for children interested to participate. A nonresponse bias may be at play in the study by Egilsson et al [26], meaning that the most motivated adolescents might have signed up to participate, resulting in lower attrition rates. From a practical point of view, we can conclude that the school is an ideal place to reach adolescents, but it may not be the right entry point for health interventions. If the intervention had been delivered through social media or through an influencer using popular youth channels such as YouTube or TikTok, it might have appealed to more adolescents [73,74]. Moreover, existing research stipulates that *health* is not a motivating factor for adolescents in health interventions [74]. Therefore, interventions that focus solely on improving health might be unlikely to engage adolescents. Rather, interventions should align with the values and priorities specified by adolescents, such as being with their friends and doing what they enjoy and are good at [74]. #LIFEGOALS was presented as an app that could motivate participants to increase healthy lifestyle behaviors. As a result, the intervention could have benefited from another framing, meaning that the current framing might not have appealed to adolescents’ motivation to use the app or their intention to change behavior (ie, no intention to change behavior=motivational phase within the Health Action Process Approach [53]). As most adolescents have only used the intervention for a short time (ie, median survival time of 10 days, 95% CI 7-17 days), it is not surprising that they could not yet experience any change as behavior change is a long-term process that usually involves several stages to ultimately bring about change [53].

Consistent with previous research, high attrition rates occurred in the very early phases of the intervention [18,26,68,75,76]: 30.1% (56/186) had only opened the app on the installation day (ie, day 1), and almost half of the adolescents (82/186, 44.1%) stopped using the app in the first week. It seems like many adolescents (approximately one-third; 56/186, 30.1%) had not given the intervention a chance. The attrition questionnaire showed that adolescents did not like the app. Despite involving the target group (ie, 249 adolescents), a graphic designer, and a retired professor passionate about software design during the development process, the numbers are not surprising, as this was still an app with research purposes. It is possible that the current generation of adolescents who have grown up with apps have much higher expectations of apps than the app presented to them as part of the study. Previous research concluded that the power of design features should not be underestimated [77]. The #LIFEGOALS app is, in that perspective, rather basic compared with existing commercial health apps, which adolescents indicated they were already using instead of the #LIFEGOALS app to track or improve their health. However, these commercial apps should be viewed with caution, as they are often not evidence-based [78]. Furthermore, previous research has shown that adolescents may assume that using health apps could make them unpopular among their peers [73], which may also have played a role in why adolescents did not like the app.

Another reason for adolescents to stop using the #LIFEGOALS app was already leading a sufficiently healthy lifestyle. However, a first glance at the baseline data from the questionnaire and the accelerometers of this sample (intervention group only) showed that 90.9% (169/186) did not reach the recommended guidelines of 60 minutes per day of moderate to vigorous physical activity, 47.8% (89/186) were sitting for >8 hours per day, 71% (132/186) did not meet the Flemish HBSC-norm of 8-hour of sleep, and 52.8% (95/180) of adolescents did not take breakfast daily. Thus, a more realistic reflection might be that adolescents have a false image of their own health behavior, overestimating themselves. Future research with this age group should focus more on the correct assessment of their own lifestyle behavior or pay more attention to communicating the guidelines, as it is unclear whether adolescents sufficiently know these.

It has been proposed that *e-attainment* may be the cause of nonadherence, which means that participants may stop using an intervention when they feel they have achieved as much as they wish from it (eg, living a sufficiently healthy life) [79-81]. In that regard, *attrition* should not always have a negative connotation. For some users and contexts, only one in-depth period of engagement with the digital intervention may be sufficient to initiate new habits or teach new skills (ie, effective engagement) [72,82]. However, this seems to be unlikely here because of the low actual use of the app components in the number of minutes. In any case, the hypothesis of e-attainment cannot be answered conclusively at this time, as the effect evaluation (in preparation) still needs to determine whether any effect of the intervention can be observed on the healthy lifestyle behaviors of adolescents.

Finally, adolescents indicated that they were not motivated by their environment to use the app. Previous research has already demonstrated that there would be a higher risk of attrition when the interventions are stand-alone apps than when they involve guidance or support [11,15-17,83]. Attrition rates to the #LIFEGOALS app could potentially be reduced if some (human) guidance or support was provided by integrating social elements [84].

Traditionally, adolescents’ SES has been measured using information about parents’ income, educational level, or occupation [85]. However, adolescents often find these measures difficult to answer [61,86]. This was confirmed here, as more than half of the adolescents indicated that they did not know the educational level of their fathers (110/186, 59.1%) and mothers (101/186, 54.3%). Furthermore, it raised the question of whether it would not be better to survey the SES of adolescents themselves rather than parental SES, as adolescence is seen as a developmental stage in which one strives to find one’s own identity, independent of one’s parents [85]. Therefore, various SES indicators were included in this study to explore whether there was a difference in attrition according to SES. In line with previous research [75,82,87,88], the results showed that adolescents’ educational level had a significant impact on attrition: attrition at any given time during the intervention period was significantly higher for adolescents from the nonacademic track compared with the academic track. The other SES indicators, family affluence and perceived financial situation, did not significantly affect attrition rates. Previous research has shown that different SES indicators have a different impact on the healthy lifestyle behaviors of adolescents [45,57,89-91]. This study shows that different SES indicators can play a different role within attrition rates as well. It is possible that the values, norms, knowledge, and skills of adolescents differ according to educational track, and that this has a greater impact on their attrition rates than their financial situation at home. Educational level is most often used as a proxy for health literacy [92], which may thus be more important for this RQ than financial resources. In this regard, surveying *cultural (health) capital* might also be an interesting SES indicator among adolescents because it maps out the values, norms, knowledge, and skills accumulated through education and lifelong socialization [92,93]. The difference in attrition according to educational level may indicate several things. First, adolescents in a nonacademic educational track may be less motivated to change health-related behavior. Second, the app (despite the integration of the narrative and chatbot) may not have been adequately tailored to the needs and preferences of adolescents in the nonacademic track [82,94]. For example, the chatbot development paper [56] showed that adolescents from the nonacademic track were involved; however, they had less input, especially during the focus groups that required some abstract thinking, than adolescents from the academic track. Therefore, we cannot say with certainty that the components adequately addressed their needs. A possible way of tailoring an intervention to people of lower affluence that has been posited in the literature is to provide a support person during the intervention period. Someone with whom they can have much more direct contact and who continues to motivate them throughout the study period, for example, by setting goals together and encouraging each other to achieve those goals [37,46]. Although this study did not find any significant differences in attrition reasons according to SES (in this study, educational track), we definitely recommend doing further (qualitative) research into this, as the number of responders from the nonacademic track was very small to make conclusive statements (12/44, 27%).

As a third RQ, this study investigated whether the duration of the 3 different intervention components during the first week had an impact on adolescents’ attrition rates and whether this differed according to SES. The results indicated that the time spent in the self-regulation and narrative components during the first week had an influence on attrition (ie, the longer time they spent in those components, the less likely they were to drop out), whereas duration in the chatbot component during the first week had no impact on the attrition rates. This may be because the chatbot could not yet answer adolescents’ questions accurately (enough), leading to user frustration and early cessation of use [56]. These links should, however, be viewed with caution, given the limited time spent in each of the components in the first week (median of 1.41, 0.03, and 0.39 minutes, respectively). Furthermore, no differences were found according to SES (ie, educational track of the adolescent), meaning that the duration use of the 3 components during the first week has the same impact on attrition for each of the two groups (academic track vs nonacademic track). At present, there is limited research within mHealth on the components that contribute to attrition. Just as it is important to investigate which mHealth components contribute to engagement [95,96], it also seems important to explore this for attrition, although participants’ engagement and attrition are undoubtedly closely linked: the stronger the engagement, the less likely it is to drop out [22].

### Limitations and Strengths

This study had some limitations. First, there was an overrepresentation of adolescents from the academic track compared with the nonacademic track in the intervention group, as well as in the respondents of the attrition questionnaire. This means that few conclusions can be drawn regarding the attrition reasons of nonacademic track adolescents. Additional research (eg, process evaluation interviews) is needed to thoroughly assess the reasons, especially in nonacademic track adolescents so that future interventions can be adopted accordingly. Second, most of our sample (128/186, 68.8%) was of medium affluence according to the Flemish HBSC cut points (mean of 9.12 on family affluence), consistent with the rather high affluence of the country [45]. This may limit the generalizability of our findings to other countries with a lower national level of affluence. Third, the last item of the FAS III regarding traveling out of Belgium for a holiday or vacation last year may be biased because of the COVID-19 pandemic and the associated travel restrictions. Fourth, no item was added to the attrition questionnaire that gauged the general motivation or need of adolescents for behavior change; therefore, we cannot say with certainty that adolescents did not use the app because they were not motivated to change their behavior. Fifth, the attrition pattern may have been influenced by sending the attrition questionnaire because the log data showed that many nonusers used the app briefly on the day they received the attrition questionnaire. Sixth, teachers did not receive specific instructions to remind or motivate adolescents to use the app during the intervention period. However, if teachers in several schools handled this differently, this might have had an impact on attrition rates. In this study, no statements could be made about this, because the specific input of the teacher, or the differences of the teachers’ input among the schools, was not questioned. The main strength is that this study added to the scarce research on attrition rates in an mHealth intervention for youth. The log data of a large group (N=186) of adolescents aged between 12 and 15 years could be tracked to gain insights into their attrition pattern. Second, SES was measured using 3 self-reported indicators. As different indicators measure different dimensions of SES, this study was able to identify which indicator plays a (greater) influence within attrition.

### Conclusions

Nonusage attrition rates in this study were high. Of the total number of adolescents, 30.1% (56/186) only opened the app on the installation day, indicating low motivation among the adolescents to use the health app. Despite the efforts made by researchers to engage low-SES adolescents, adolescents from a nonacademic educational track were more likely to drop out earlier than adolescents from an academic track. The reasons for attrition greatly varied. Duration in the self-regulation and narrative components during the first week may have a positive impact on attrition rates, both for adolescents in academic and nonacademic educational tracks.

## Appendix

Multimedia Appendix 1 Flowchart of the #LIFEGOALS intervention.

Multimedia Appendix 2 Screenshot of the #LIFEGOALS app.

Multimedia Appendix 3 Roll-up banner.

Multimedia Appendix 4 Results of the standard Cox proportional hazard regression models (without clustering).

Multimedia Appendix 5 Reasons why adolescents stopped using the #LIFEGOALS app.

## Abbreviations

FAS: Family Affluence Scale
HBSC: Health Behavior in School-Aged Children
mHealth: mobile health
RQ: research question
SES: socioeconomic status

## Data Availability

The data sets used or analyzed during this study are available from the corresponding author upon reasonable request.
