# Supplementary figures and images for: Nonusage Attrition of Adolescents in an mHealth Promotion Intervention and the Role of Socioeconomic Status: Secondary Analysis of a 2-Arm Cluster-Controlled Trial
Source: JMIR Mhealth Uhealth. 2022 May 10;10(5):e36404. doi: 10.2196/36404 (PMC9131163; doi:10.2196/36404)

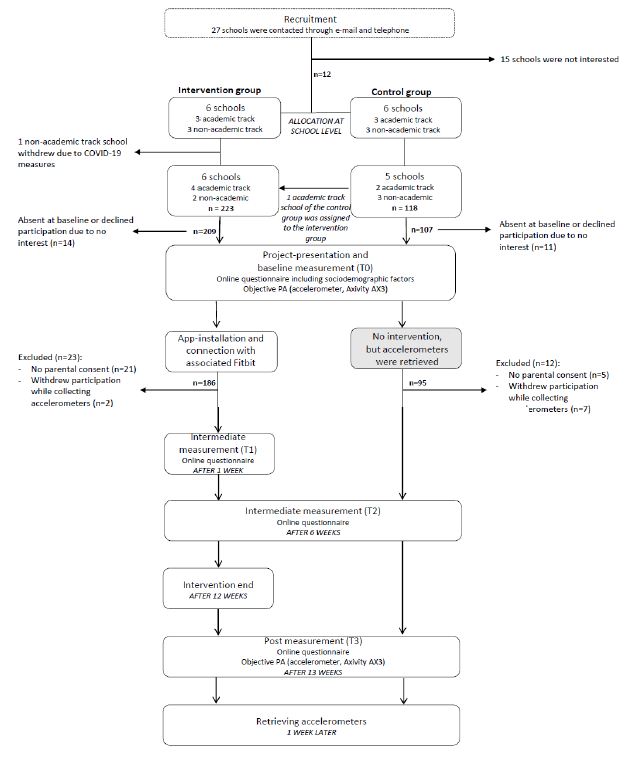

Supplement: Multimedia Appendix 1 [file mhealth_v10i5e36404_app1.png]

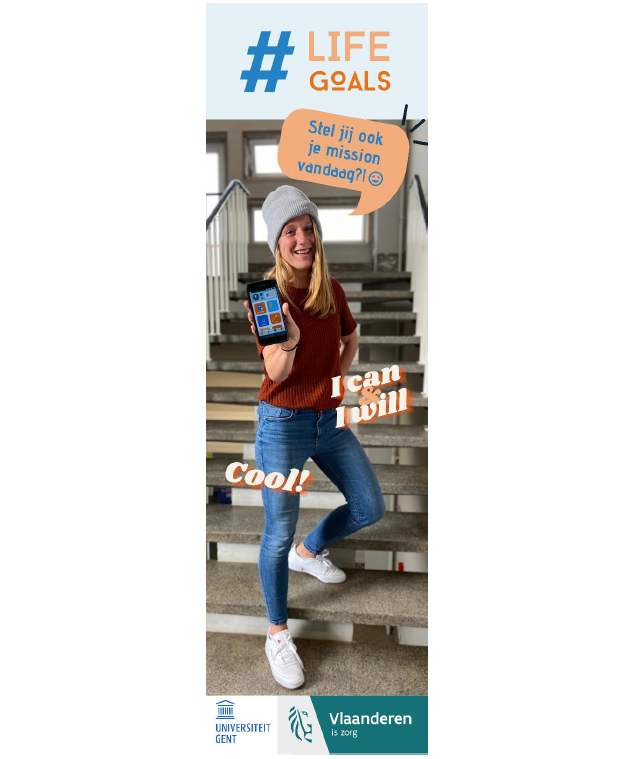

Supplement: Multimedia Appendix 3 [file mhealth_v10i5e36404_app3.png]
