# Supplementary material for: Nonusage Attrition of Adolescents in an mHealth Promotion Intervention and the Role of Socioeconomic Status: Secondary Analysis of a 2-Arm Cluster-Controlled Trial
Source: JMIR Mhealth Uhealth. 2022 May 10;10(5):e36404. doi: 10.2196/36404 (PMC9131163; doi:10.2196/36404)
Supplement: Multimedia Appendix 2 [file mhealth_v10i5e36404_app2.pdf]

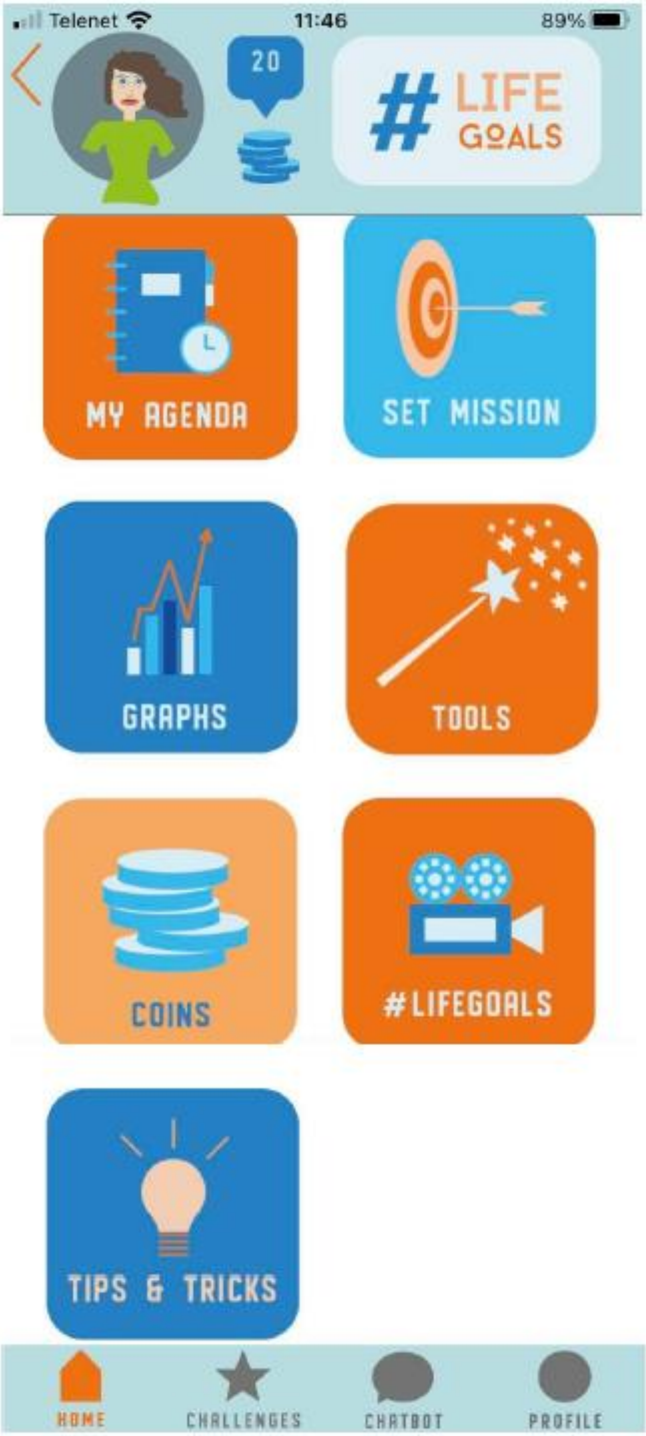

Telenet
11:46
89%
20
LIFE GOALS
MY AGENDA
SET MISSION
GRAPHS
TOOLS
COINS
#LIFEGOALS
TIPS & TRICKS
HOME
CHALLENGES
CHATBOT
PROFILE

The self-regulation components:

- Set Mission: goal setting and action planning
- My Agenda: goal setting and action planning
- Tools: coping planning
- Graphs: self-monitoring

The narrative:

- #LIFEGOALS

The chatbot:

- Chatbot (in the bottom bar)

Information on the benefits of health behaviors and on relevant (youth) health organizations for further information or support:

- Tips and Tricks

Reward system:

- Coins
- Challenges (to earn extra coins)
- Avatar (left in the upper corner, by clicking on it they could further pimp the avatar)
