# Supplementary material for: Nonusage Attrition of Adolescents in an mHealth Promotion Intervention and the Role of Socioeconomic Status: Secondary Analysis of a 2-Arm Cluster-Controlled Trial
Source: JMIR Mhealth Uhealth. 2022 May 10;10(5):e36404. doi: 10.2196/36404 (PMC9131163; doi:10.2196/36404)
Supplement: Multimedia Appendix 4 [file mhealth_v10i5e36404_app4.docx]

**Multimedia Appendix 4**: Results of the standard Cox proportional hazard regression models (without clustering)

|  | **Single-predictor models** | | | **Multiple-predictor models** | | | | | |
| --- | --- | --- | --- | --- | --- | --- | --- | --- | --- |
|  |  | | | **Without interaction term** | | | **With interaction term** | | |
|  | **Coefficient (SE)** | **HR  (95% CI)** | ***P*-value** | **Coefficient (SE)** | **HR**  **(95% CI)** | ***P*-value** | **Coefficient (SE)** | **HR**  **(95% CI)** | ***P*-value** |
| **Socio-demographic variables** | | | | | | | | | |
| Age | -0.003 (0.080) | 0.997  (0.852, 1.167) | .97 | - | - | - | - | - | - |
| Gender (ref. boy)   - Girl - Other | -0.391 (0.154)  0.338 (0.717) | 0.677 (0.501, 0.915)  1.402 (0.344, 5.708) | *.011*  .637 | -0.198 (0.160)  0.313 (0.729) | 0.820  (0.600, 1.122) 1.367 (0.328, 5.704) | .215  .668 | -0.220 (0.167)  0.340 (0.731) | 0.802 (0.579, 1.112)  1.405 (0.336, 5.887) | .186  .642 |
| Grade (ref. 7^th^ grade)   - 8^th^ grade - 9^th^ grade | -0.060 (0.182)  -0.231 (0.183) | 0.942  (0.659, 1.347)  0.794 (0.554, 1.137) | .744  .208 | -  - | -  - | -  - | -  - | -  - | -  - |
| Home language (ref. Dutch) | 0.518 (0.186) | 1.678 (1.165, 2.416) | *.005* | 0.109 (0.196) | 1.115 (0.760, 1.636) | .578 | 0.102 (0.197) | 1.107 (0.752, 1.630) | .606 |
| Educational track (ref. academic track) | 0.630 (0.159) | 1.878 (1.376, 2.563) | *<.001* | 0.698 (0.177) | 2.009 (1.419, 2.844) | *<.001* | 0.745 (0.228) | 2.106 (1.348, 3.293) | *.001* |
| Family affluence (ref. low FAS)   - Medium FAS - High FAS | -0.340 (0.190)  0.069 (0.284) | 0.711 (0.490, 1.033)  (0.614, 1.868) | .074  .808 | - | - | - | - | - | - |
| Perceived financial situation | -0.219 (0.187) | 0.804 (0.557, 1.160) | .243 | - | - | - | - | - | - |
| **Log data-derived variables** | | | | | | | | | |
| Duration self-regulation during first week | -0.100 (0.017) | 0.905 (0.875, 0.937) | *<.001* | -0.100 (0.020) | 0.905 (0.870, 0.941) | *<.001* | -0.094 (0.021) | 0.911 (0.875, 0.948) | *<.001* |
| Duration narrative during first week | -0.088 (0.036) | 0.915 (0.853, 0.983) | *.015* | -0.073 (0.036) | 0.929 (0.865, 0.998) | *.044* | -0.026 (0.044) | 0.975 (0.894, 1.063) | .561 |
| Duration chatbot during first week | -0.061 (0.027) | 0.941 (0.893, 0.993) | *.025* | -0.010 (0.028) | 1.010 (0.956, 1.066) | .724 | -0.017 (0.042) | 0.983 (0.906, 1.067) | .683 |
| **Interaction with SES** | | | | | | | | | |
| Duration self-regulation during first week*Educational track (ref. academic track) | - | - | - | - | - | - | -0.012 (0.045) | 0.989 (0.905, 1.079) | .797 |
| Duration narrative during first week *Educational track (ref. academic track) | - | - | - | - | - | - | -0.104 (0.080) | 0.902 (0.771, 1.055) | .195 |
| Duration chatbot during first week*Educational track (ref. academic track) | - | - | - | - | - | - | 0.046 (0.057) | 1.047 (0.937, 1.170) | .422 |
