# Supplementary material for: Nonusage Attrition of Adolescents in an mHealth Promotion Intervention and the Role of Socioeconomic Status: Secondary Analysis of a 2-Arm Cluster-Controlled Trial
Source: JMIR Mhealth Uhealth. 2022 May 10;10(5):e36404. doi: 10.2196/36404 (PMC9131163; doi:10.2196/36404)
Supplement: Multimedia Appendix 5 [file mhealth_v10i5e36404_app5.pdf]

REASONS WHY ADOLESCENTS STOPPED USING THE #LIFEGOALS-APP

I do not use the #LIFEGOALS app anymore because… (n=44)	Strongly disagree		Disagree		Neutral		Agree		Strongly agree	
**the app takes too much time**	**n=6**	**13.6%**	**n=9**	**20.5%**	**n=16**	**36.4%**	**n=7**	**15.9%**	**n=6**	**13.6%**
Academic track	*n=5*	*15.6%*	*n=7*	*21.9%*	*n=12*	*37.5%*	*n=4*	*12.5%*	*n=4*	*12.5%*
Non-academic track	*n=1*	*8.3%*	*n=2*	*16.7%*	*n=4*	*33.3%*	*n=3*	*25.0%*	*n=2*	*16.7%*
**I am not allowed to use my mobile phone much at home**	**n=23**	**52.3%**	**n=9**	**20.5%**	**n=7**	**15.9%**	**n=5**	**11.4%**	**n=0**	**0.0%**
Academic track	*n=16*	*50.0%*	*n=7*	*21.9%*	*n=5*	*15.6%*	*n=4*	*12.5%*	*n=0*	*0.0%*
Non-academic track	*n=7*	*58.3%*	*n=2*	*16.7%*	*n=2*	*16.7%*	*n=1*	*8.3%*	*n=0*	*0.0%*
**I already live a sufficiently healthy life**	**n=2**	**4.5%**	**n=4**	**9.1%**	**n=22**	**50.0%**	**n=10**	**22.7%**	**n=6**	**13.6%**
Academic track	*n=0*	*0.0%*	*n=4*	*12.5%*	*n=16*	*50.0%*	*n=7*	*21.9%*	*n=5*	*15.6%*
Non-academic track	*n=2*	*16.7%*	*n=0*	*0.0%*	*n=6*	*50.0%*	*n=3*	*25.0%*	*n=1*	*8.3%*
**there were technical problems with the app**	**n=21**	**47.7%**	**n=8**	**18.2%**	**n=6**	**13.6%**	**n=4**	**9.1%**	**n=5**	**11.4%**
Academic track	*n=14*	*43.8%*	*n=6*	*18.8%*	*n=4*	*12.5%*	*n=3*	*9.4%*	*n=5*	*15.6%*
Non-academic track	*n=7*	*58.3%*	*n=2*	*16.7%*	*n=2*	*16.7%*	*n=1*	*8.3%*	*n=0*	*0.0%*
**the app was too complicated**	**n=11**	**25.0%**	**n=10**	**22.7%**	**n=12**	**27.3%**	**n=10**	**22.7%**	**n=1**	**2.3%**
Academic track	*n=9*	*28.1%*	*n=8*	*25.0%*	*n=9*	*28.1%*	*n=6*	*18.8%*	*n=0*	*0.0%*
Non-academic track	*n=2*	*16.7%*	*n=7*	*16.7%*	*n=3*	*25.0%*	*n=4*	*33.3%*	*n=1*	*8.3%*
**I did not like the app**	**n=4**	**9.1%**	**n=6**	**13.6%**	**n=17**	**38.6%**	**n=13**	**29.5%**	**n=4**	**9.1%**
Academic track	*n=3*	*9.4%*	*n=3*	*9.4%*	*n=13*	*40.6%*	*n=11*	*34.4%*	*n=2*	*6.3%*
Non-academic track	*n=1*	*8.3%*	*n=3*	*25.0%*	*n=4*	*33.3%*	*n=2*	*16.7%*	*n=2*	*16.7%*
**my behavior did not change by using the app**	**n=4**	**9.1%**	**n=5**	**11.4%**	**n=11**	**25.0%**	**n=12**	**27.3%**	**n=12**	**27.3%**
Academic track	*n=3*	*9.4%*	*n=5*	*15.6%*	*n=8*	*25.0%*	*n=9*	*28.1%*	*n=7*	*21.9%*
Non-academic track	*n=1*	*8.3%*	*n=0*	*0.0%*	*n=3*	*25.0%*	*n=3*	*25.0%*	*n=5*	*41.7%*
**the app did not meet my expectations**	**n=4**	**9.1%**	**n=15**	**34.1%**	**n=18**	**40.9%**	**n=4**	**9.1%**	**n=3**	**6.8%**
Academic track	*n=2*	*6.3%*	*n=11*	*34.4%*	*n=13*	*40.6%*	*n=4*	*12.5%*	*n=2*	*6.3%*
Non-academic track	*n=2*	*16.7%*	*n=4*	*33.3%*	*n=5*	*41.7%*	*n=0*	*0.0%*	*n=1*	*8.3%*
**my friends did not use the app either**	**n=9**	**20.5%**	**n=13**	**29.5%**	**n=15**	**34.1%**	**n=5**	**11.4%**	**n=2**	**4.5%**
Academic track	*n=8*	*25.0%*	*n=9*	*28.1%*	*n=11*	*34.4%*	*n=2*	*6.3%*	*n=2*	*6.3%*
Non-academic track	*n=1*	*8.3%*	*n=4*	*33.3%*	*n=4*	*33.3%*	*n=3*	*25.0%*	*n=0*	*0.0%*

**I did not get enough reminders to use the app**	**n=8**	**18.2%**	**n=11**	**25.0%**	**n=15**	**34.1%**	**n=8**	**18.2%**	**n=2**	**4.5%**
Academic track	*n=3*	*9.4%*	*n=8*	*25.0%*	*n=12*	*37.5%*	*n=7*	*21.9%*	*n=2*	*6.3%*
Non-academic track	*n=5*	*41.7%*	*n=3*	*25.0%*	*n=3*	*25.0%*	*n=1*	*8.3%*	*n=0*	*0.0%*
**I was not motivated by my environment to keep using the app (e.g. at home, by friends, etc.)**	**n=4**	**9.1%**	**n=7**	**15.9%**	**n=18**	**40.9%**	**n=10**	**22.7%**	**n=5**	**11.4%**
Academic track	*n=2*	*6.3%*	*n=3*	*9.4%*	*n=14*	*43.8%*	*n=10*	*31.3%*	*n=3*	*9.4%*
Non-academic track	*n=6*	*50.0%*	*n=1*	*8.3%*	*n=1*	*8.3%*	*n=2*	*16.7%*	*n=2*	*16.7%*
**I already use other apps to track and/or improve my lifestyle (e.g. Fitbit app)**	**n=12**	**27.3%**	**n=5**	**11.4%**	**n=10**	**22.7%**	**n=12**	**27.3%**	**n=5**	**11.4%**
Academic track	*n=6*	*18.8%*	*n=4*	*12.5%*	*n=9*	*28.1%*	*n=10*	*31.3%*	*n=3*	*9.4%*
Non-academic track	*n=6*	*50.0%*	*n=1*	*8.3%*	*n=1*	*8.3%*	*n=2*	*16.7%*	*n=2*	*16.7%*
**there are other things in my life I consider more important than my health**	**n=6**	**13.6%**	**n=13**	**29.5%**	**n=18**	**40.9%**	**n=5**	**11.4%**	**n=2**	**4.5%**
Academic track	*n=4*	*12.5%*	*n=11*	*34.4%*	*n=14*	*43.8%*	*n=3*	*9.4%*	*n=0*	*0.0%*
Non-academic track	*n=2*	*16.7%*	*n=2*	*16.7%*	*n=4*	*33.3%*	*n=2*	*16.7%*	*n=2*	*16.7%*
**the chatbot often answered my questions incorrectly**	**n=14**	**31.8%**	**n=8**	**18.2%**	**n=16**	**36.4%**	**n=6**	**13.6%**	**n=0**	**0.0%**
Academic track	*n=9*	*28.1%*	*n=7*	*21.9%*	*n=12*	*37.5%*	*n=4*	*12.5%*	*n=0*	*0.0%*
Non-academic track	*n=5*	*41.7%*	*n=1*	*8.3%*	*n=4*	*33.3%*	*n=2*	*16.7%*	*n=0*	*0.0%*
